# Preeclampsia and Site-Specific Cancer Risk: A Nationwide Population-Based Study

**DOI:** 10.3390/cancers18142218

**Published:** 2026-07-09

**Authors:** Hyewon Hur, Eun Hwa Kim, Myeongjee Lee, Inkyung Jung, Kyung Jin Eoh

**Affiliations:** 1 Department of Obstetrics and Gynecology, Yonsei University College of Medicine, Yongin Severance Hospital, Yongin 16995, Republic of Korea; hwhur201@yuhs.ac; 2 Institute of Women’s Life Medical Science, Yonsei University College of Medicine, Seoul 03722, Republic of Korea; 3 Biostatistics Collaboration Unit, Department of Biomedical Systems Informatics, Yonsei University College of Medicine, Seoul 03722, Republic of Korea; ehkim0607@yuhs.ac (E.H.K.); mlee1004@yuhs.ac (M.L.); 4 Division of Biostatistics, Department of Biomedical Systems Informatics, Yonsei University College of Medicine, Seoul 03722, Republic of Korea

**Keywords:** preeclampsia, gallbladder and biliary tract cancer, breast cancer, thyroid cancer

## Abstract

**Background:** Preeclampsia, a condition of high blood pressure during pregnancy, is linked to microangiopathy in various organs and may contribute to cancer development. This study aimed to evaluate cancer risk in women with newly diagnosed preeclampsia. **Methods:** We conducted a nationwide, population-based retrospective study using data from the Korean National Health Insurance claims database (2008–2020). Women diagnosed with preeclampsia between 2009 and 2013 were compared to a control group who underwent appendectomy but did not have preeclampsia. Participants with a prior cancer diagnosis were excluded. Cancer occurrence was assessed using the International Classification of Diseases, 10th revision codes. **Results:** Data from 42,380 preeclampsia patients and 105,327 controls were analyzed. Cancer incidence rates were 333.1 per 100,000 person-years in the preeclampsia group and 377.0 in controls. Preeclampsia was associated with significantly higher risks of gallbladder and biliary tract cancers (HR 5.49, 95% CI 1.23–24.50), breast cancer (HR 1.17, 95% CI 1.02–1.34), and thyroid cancer (HR 1.21, 95% CI 1.10–1.34). However, it was linked to lower risks of ovarian cancer (HR 0.44, 95% CI 0.27–0.74) and leukemia (HR 0.36, 95% CI 0.16–0.81). All hazard ratios were adjusted for age, which differed substantially between the two groups; unadjusted incidence rates and age-adjusted hazard ratios therefore differed in direction for some sites. **Conclusions:** Preeclampsia was associated with an increased risk of certain cancers, including breast and thyroid cancers, and with a decreased risk of ovarian cancer and leukemia. Because the control group was older than the preeclampsia group, crude incidence rates and age-adjusted hazard ratios differed in direction; the age-adjusted estimates should therefore be regarded as the primary findings. Several site-specific associations, particularly those based on small event counts (e.g., gallbladder and biliary tract cancer), should be interpreted as exploratory and warrant replication.

## Simple Summary

Preeclampsia is a serious blood-pressure disorder affecting roughly 2–8% of pregnancies, and it disturbs blood vessels, angiogenic signaling, and hormones in ways that may persist long after delivery. Whether these lasting changes raise a woman’s future cancer risk has remained unclear, particularly for cancers beyond the breast. Using a nationwide Korean National Health Insurance database, we compared long-term cancer incidence between women with a history of preeclampsia and a comparison group followed over more than a decade. Women with prior preeclampsia showed an elevated risk of several cancers—including gallbladder and biliary tract cancer, reported here for the first time, alongside breast and thyroid cancer—while their risk of ovarian cancer and leukemia appeared lower. The increased risk was most concentrated among younger women aged 20–29. These findings suggest that a history of preeclampsia may help identify women who could benefit from earlier or closer cancer surveillance, and they open new avenues for understanding how pregnancy complications shape long-term health.

## 1. Introduction

Preeclampsia is a pregnancy-specific disorder characterized by hypertension and proteinuria, estimated to affect 2–8% of pregnancies [1]. Despite ongoing research, the underlying pathophysiology of preeclampsia remains largely unknown. However, the clinical manifestations of preeclampsia have been linked to microangiopathy in various target organs, likely resulting from extensive dysfunction of maternal endothelial cells [2,3].

Considering that dysfunctional endothelial cells are active contributors to the tumor microenvironment and the efficacy of anti-angiogenic therapies in multiple cancers, preeclampsia may cause changes in the endothelium and arteries in pregnant women, potentially influencing their future cancer risks [4,5,6]. Moreover, cancer is often closely related to levels of blood hormones such as estrogen, progesterone, androgen, human chorionic gonadotropin, and insulin-like growth factor-1, which are affected by preeclampsia [7,8].

Although several systematic reviews have indicated a potential association between preeclampsia and breast cancer risk, the conclusions drawn from these studies have been inconsistent [8,9,10]. Furthermore, the relationship between preeclampsia and the risks of other types of cancer remains an area of ongoing research and is not yet well understood.

This study hypothesizes that preeclampsia may correlate to an increased cancer risk later in life. To explore this potential association, we conducted a nationwide population-based study using data from the Korean National Health Insurance (NHI) 10-year database.

## 2. Methods

### 2.1. Study Design and Database

A retrospective study was conducted using data from the NHI claims database from January 2008 to August 2020. The data were accessed for research purposes on 23 September 2021. The NHI is a compulsory scheme in Republic of Korea that covers an estimated 51 million citizens, providing various healthcare services such as pharmaceuticals, outpatient and inpatient care, and diagnostic and surgical procedures. It operates as a single-payer system funded by a combination of employee and employer contributions, along with government subsidies. The Health Insurance Review and Assessment Service (HIRA) manages the comprehensive database of all medical treatments and services provided to patients, along with their demographic information and diagnoses coded according to the International Classification of Diseases, 10th revision (ICD-10). All medical institutions are required to report this information to HIRA for reimbursement purposes.

### 2.2. Study Population Identification

Women aged 20–49 years were included as the study population. The data were obtained from the NHI claims database, and the study participants were selected based on their previous diagnosis of preeclampsia (ICD-10 code: O14) and consultations with a physician between 2009 and 2013. To be eligible for the study, the participants must not have had any cancer diagnosis before their preeclampsia diagnosis.

For the control group, women aged 20–49 years who underwent an appendectomy between 2009 and 2013 were selected. Appendectomy patients were selected as the reference group for several methodological reasons. First, acute appendicitis is an unplanned, physiologically acute condition with no established etiological association with any of the cancers examined in this study, thereby minimizing the risk of introducing systematic cancer-directional bias through control selection. Second, because both preeclampsia patients and appendectomy patients actively sought medical care and received treatment within the universal NHI system, the two groups share comparable healthcare-seeking behavior and equivalent access to cancer surveillance, reducing the likelihood of differential detection bias. Third, restricting the control group to women who had undergone a surgical procedure ensured that all participants had been evaluated by a physician at least once during the index period, creating a symmetrical opportunity for concurrent cancer detection. Women in the control group were excluded if they had received any medical consultation for preeclampsia during the study period or had a prior cancer diagnosis. The index date for each group was either the initial diagnosis of preeclampsia or the date of the appendectomy, as recorded in the HIRA database. By design, the surgical procedure (appendectomy) was an inclusion criterion for the control group only; women in the preeclampsia group were identified solely on the basis of their clinical diagnosis (ICD-10 code O14) and were not required to have undergone any surgical procedure. The two groups were therefore distinguished by their respective index events (a clinical diagnosis vs. a surgical procedure), and no surgical or chemotherapeutic treatment was imposed on the preeclampsia group as part of study population selection.

### 2.3. Diagnosis of Cancer

The study identified cancer cases in the two groups using the ICD-10 diagnostic codes for various cancer types based on different organ sites. These codes included: C01–C14 for oral cavity and pharynx, C15 for esophagus, C16 for stomach, C18–C20 for colon and rectum, C21 for anus and anal canal, C22 for liver, C23 and C24 for gallbladder and biliary tract, C25 for pancreas, C30–C32 for nasal cavity and larynx, C33 and C34 for lung, C37–C39 and C45 for thorax, C40 and C41 for bone, C48 for retroperitoneum and peritoneum, C50 for breast, C51–C53 for cervix, C54 and C55 for uterus, C56 for ovary, C64 and C65 for kidney, C66 and C67 for bladder, C70–C72 for brain and spinal cord, C73 for thyroid, C81 for Hodgkin lymphoma, C82–C85 and C96 for non-Hodgkin lymphoma, C90 for multiple myeloma, C91–C95 for leukemia, and C00, C17, C26, C43, C44, C46, C47, C49, C57, C58, C68, C69, C74–C76, C77–C80, C86, C88, and C97 for other cancer types.

The South Korean government provides financial aid to patients with rare, incurable diseases such as cancer. These patients are identified with a specific code for “exempted calculation of health insurance” based on a clinicopathological evaluation. Thus, using cancer-related ICD-10 codes with the additional code (V193 or V194) for rare incurable diseases to identify patients with cancer was considered reliable. Cancer occurrence was defined as having at least two visits to a physician for reasons related to the corresponding diagnostic code. The study calculated both the overall and organ-specific cancer risks in both groups.

### 2.4. Confounding Factors

The study aimed to identify potential confounding variables such as age and comorbidities, previously reported to be associated with cancer incidence [11]. Comorbidities were chosen based on their known association with cancer. The following conditions were considered: diabetes (ICD-10 codes: E10, E11, E12, E13, and E14), hyperlipidemia (E78), hypertension (I10), heart failure (I50), chronic obstructive pulmonary disease (J44), chronic liver cirrhosis (K74 and K703), and kidney disease (N18). Patients with at least two visits with the corresponding diagnostic code within 1 year before the index date were considered to have concurrent comorbidity.

### 2.5. Statistical Analyses

The mean and standard deviation are used to report continuous variables, and an independent two-sample unpaired two-tailed *t*-test was performed to compare continuous variables between the preeclampsia and control groups, assuming approximate normality of the underlying distributions given the large sample size. Categorical variables are presented as numbers (%) and were compared using the chi-square or Fisher’s exact tests. To estimate the incidence rate ratio (IRR) of cancer in patients with preeclampsia and controls, we performed a Poisson regression analysis and used an offset for person-years. To assess the risk of cancer development after adjusting for confounding variables, we used a multivariate Cox proportional hazards model to calculate the hazard ratio (HR). All statistical tests were two-tailed, and statistical significance was set at *p* < 0.05. All analyses were performed using SAS Enterprise Guide version 7.1 (SAS Institute Inc., Cary, NC, USA; RRID:SCR_008567) and R version 3.5.1 (R Foundation for Statistical Computing; RRID:SCR_001905). To assess the robustness of the primary findings across the reproductive age spectrum, age-stratified analyses were performed as pre-specified sensitivity analyses. Participants were stratified into three age subgroups (20–29 years, 30–39 years, and 40–49 years), defined by age at the index date (i.e., at preeclampsia diagnosis or appendectomy, not at cancer diagnosis), and overall cancer risk and organ-specific cancer incidence rates were re-estimated in each stratum using the same multivariate Cox proportional hazards model and Poisson regression approach applied in the primary analysis. No formal a priori power calculation was performed, as this was a population-based study utilizing all eligible patients identified in the nationwide NHI claims database during the index period; the resulting sample size (*n* = 147,707) was considered sufficient to detect clinically meaningful differences in cancer risk. Because this is a retrospective cohort study using administrative claims data, group assignment was not randomized; individuals were classified into the preeclampsia or control group based on their recorded diagnoses and procedures in the HIRA database, as described above. Blinding of investigators to group assignment was not applicable in this retrospective database study; however, outcome ascertainment was performed objectively using pre-specified ICD-10 diagnostic codes and government-issued cancer exemption codes (V193/V194), without investigator judgment, thereby minimizing the potential for differential outcome misclassification. Participants were followed from the index date until the occurrence of cancer, death, or the end of the study period (August 2020), whichever came first. Follow-up duration was quantified both as person-years and as the mean number of follow-up years per group; because index dates spanned 2009–2013 and follow-up ended in August 2020, per-participant follow-up ranged from approximately 6.7 to 11.6 years (mean 9.06 years in each group). Loss to follow-up was not applicable in this study, as the NHI database captures all healthcare encounters for the entire enrolled population; however, participants who died before developing cancer were censored at the date of death, and death before the last follow-up date was recorded for 186 controls (0.18%) and 100 preeclampsia patients (0.24%).

### 2.6. Study Approval

The study adhered to the ethical standards established in the Declaration of Helsinki. The Institutional Review Board (IRB) of Yongin Severance Hospital, Yonsei University College of Medicine (Yongin, Korea) reviewed and approved the study protocol. The IRB waived the need for obtaining informed consent from the participants due to the study’s retrospective nature (approval number: 9-2020-0088; date of approval: 31 August 2020).

## 3. Results

### 3.1. Study Population

**Figure 1:**
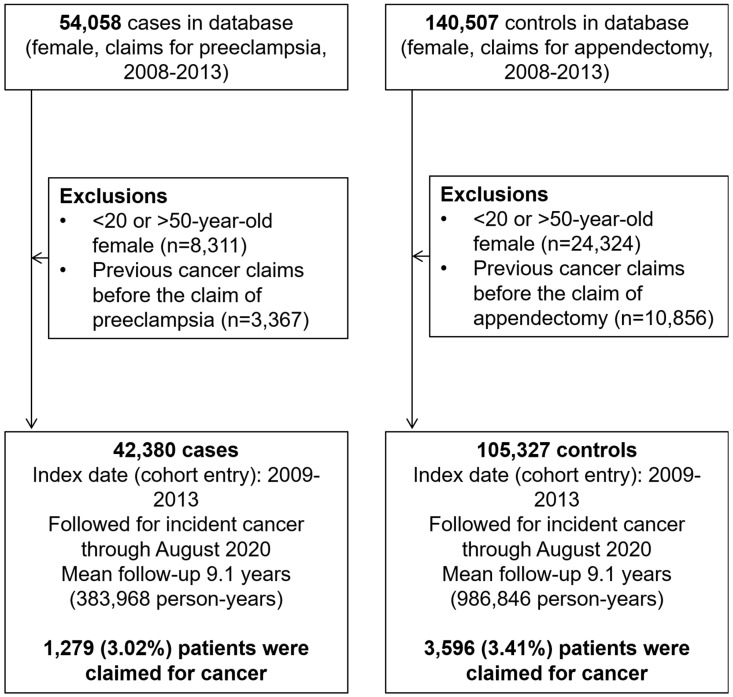
Flow diagram of the study population of cases and controls. Women with claims for preeclampsia and appendectomy controls were identified from the National Health Insurance database (claims identification 2008–2013) and entered follow-up at the index date (2009–2013). Both arms were followed for incident cancer through August 2020 (mean follow-up approximately 9.1 years; 383,968 person-years in the preeclampsia arm and 953,846 in the control arm). Cancer was ascertained by at least two physician visits with the relevant ICD-10 and government cancer-exemption codes (V193/V194); all counted cancers were incident. Because analyses were conducted within the HIRA closed research environment, from which only aggregate outputs were exported, a patient-level summary of the interval from index date to diagnosis was not available for reporting; the incident, post-index case definition nonetheless guarantees the correct temporal sequence.

This study included 42,380 patients with preeclampsia and 105,327 controls (Figure 1). Before adjustment, the preeclampsia group had a significantly younger age, higher socioeconomic status, and higher rates of comorbidities, including diabetes, hypertension, chronic kidney disease, and heart failure than the control group (Table 1).

### 3.2. Cancer Incidence and Site

**Figure 2:**
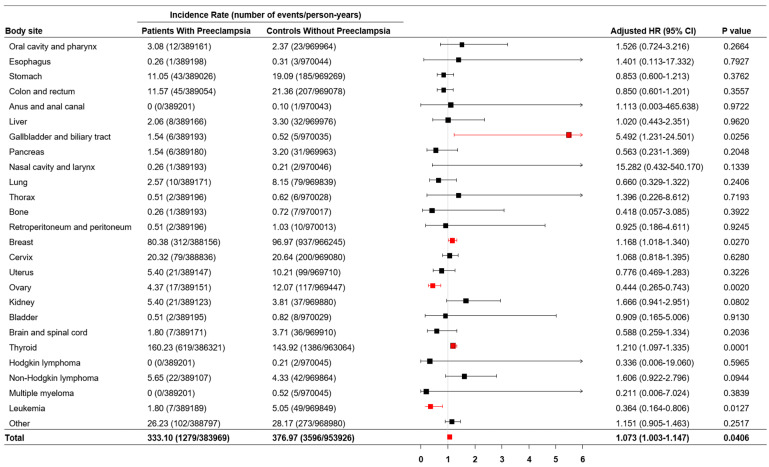
Incidence rates and adjusted hazard ratios.

Participants were followed from the index date (preeclampsia diagnosis or appendectomy, occurring between 2009 and 2013) until cancer diagnosis, death, or the end of the study period (August 2020), whichever came first, so that individual follow-up ranged from approximately 6.7 to 11.6 years. Mean follow-up was essentially identical between arms—9.06 years in the preeclampsia group (383,968 person-years) and 9.06 years in the control group (953,846 person-years)—indicating that time at risk was balanced between the groups and did not differentially affect cancer ascertainment in either arm. All counted cancers were incident and ascertained after the index date across the full observation window. The incidence rates of cancer were 377.0 per 100,000 person-years in the control group and 333.1 per 100,000 person-years in the preeclampsia group (IRR, 0.88; 95% confidence interval [CI], 0.83–0.94) (Figure 2). Patients with preeclampsia had significantly decreased risks of cancer of the stomach (IRR, 0.58; 95% CI, 0.42–0.81; *p* < 0.001), colon and rectum (IRR, 0.54; 95% CI, 0.39–0.75; *p* < 0.001), lung (IRR, 0.32; 95% CI, 0.16–0.61; *p* < 0.001), breast (IRR, 0.83; 95% CI, 0.73–0.94; *p* = 0.004), uterus (IRR, 0.53; 95% CI, 0.33–0.85; *p* = 0.007), ovary (IRR, 0.36; 95% CI, 0.22–0.60; *p* < 0.001), and leukemia (IRR, 0.36; 95% CI, 0.16–0.79; *p* = 0.0076), compared with the control group. Notably, an increased risk was observed for thyroid cancer (IRR, 1.11; 95% CI, 1.01–1.22; *p* = 0.026) (Figure 2 and Table 2).

Moreover, in univariate and multivariate Cox proportional hazards analyses of the cancer risks, preeclampsia and age were significant predictive factors for cancer diagnosis (Table 3). Patients with preeclampsia had a significantly increased risk of cancers of the gallbladder and biliary tract (HR, 5.49; 95% CI, 1.23–24.50; *p* = 0.026), breast (HR, 1.17; 95% CI, 1.02–1.34; *p* = 0.027), and thyroid (HR, 1.21; 95% CI, 1.10–1.34; *p* < 0.001). Conversely, a significantly decreased risk was observed for ovarian cancer (HR, 0.44; 95% CI, 0.26–0.74; *p* = 0.002) and leukemia (HR, 0.36; 95% CI, 0.16–0.81; *p* = 0.013).

For several sites, most notably breast cancer, the crude incidence rate ratios and the age-adjusted hazard ratios point in opposite directions (e.g., breast cancer IRR 0.83 vs. adjusted HR 1.17). This apparent discrepancy is attributable to the substantial age difference between the two groups. Because the preeclampsia group was considerably younger than the control group (only 4.7% vs. 27.9% were aged 40–49 years) and cancer incidence rises steeply with age (adjusted HR 1.06 per year), the crude rates are dominated by the age structure and understate the age-adjusted association. Once age is accounted for in the multivariable Cox model, the direction of association reverses; the age-adjusted hazard ratios are therefore the appropriate effect estimates and are the basis for the conclusions below.

In the age-stratified sensitivity analyses, the overall cancer risk associated with preeclampsia differed substantially by age group (Supplementary Table S1). Among women aged 20–29 years, preeclampsia was associated with a significantly elevated overall cancer risk (adjusted HR, 1.217; 95% CI, 1.055–1.403; *p* = 0.007), a finding that was more pronounced than the primary all-age estimate (adjusted HR, 1.073; 95% CI, 1.003–1.147; *p* = 0.041). By contrast, no statistically significant association between preeclampsia and overall cancer risk was observed among women aged 30–39 years (adjusted HR, 0.950; 95% CI, 0.871–1.036; *p* = 0.248) or 40–49 years (adjusted HR, 0.910; 95% CI, 0.743–1.113; *p* = 0.358), suggesting that the cancer risk conferred by preeclampsia may be most clinically relevant in the youngest reproductive age group. Site-specific incidence rate analyses within each stratum were directionally consistent with the primary findings. The elevated incidence of thyroid cancer in preeclampsia patients was most pronounced in the 20–29-year subgroup (incidence rate, 131.64 [95% CI, 112.09–153.62] per 100,000 person-years in the preeclampsia group vs. 90.26 [95% CI, 80.66–100.70] in controls; *p* = 0.0003), whereas the difference was not statistically significant in women aged 30–39 years (178.15 [95% CI, 161.80–195.70] vs. 163.82 [95% CI, 150.42–178.09]; *p* = 0.194). The protective association with ovarian cancer was most evident in the 30–39-year group, in which preeclampsia patients had a markedly lower incidence rate compared with controls (3.69 [95% CI, 1.69–7.00] vs. 11.00 [95% CI, 7.75–15.16] per 100,000 person-years; *p* = 0.0008). Similarly, the reduced leukemia incidence in preeclampsia patients was statistically significant among women aged 20–29 years (0.82 [95% CI, 0.02–4.56] vs. 4.22 [95% CI, 2.36–6.96] per 100,000 person-years; *p* = 0.013) but not in the older age strata, consistent with the primary analysis.

## 4. Discussion

In this nationwide Korean population-based study (*n* = 147,707), preeclampsia had a significant impact on the risks of gallbladder and biliary tract cancer (HR, 5.49), breast cancer (HR, 1.17), thyroid cancer (HR, 1.21), ovarian cancer (HR, 0.44), and leukemia (HR, 0.36).

A diagnosis of preeclampsia was significantly associated with an increased incidence of several types of cancer, including gallbladder, biliary tract, breast, and thyroid cancer. Specifically, the risk of gallbladder and biliary tract cancer was significantly elevated in women with preeclampsia. The underlying mechanism for this association is not yet fully understood. However, right upper abdominal pain is a common symptom of preeclampsia. It is thought to be related to gallbladder wall thickening caused by subserosal edema due to increased vascular permeability and cardiac dysfunction, similar to the changes observed in other organs in preeclampsia [12]. Additionally, it has been established that pregnant women are at an increased risk of developing cholelithiasis and cholecystitis. This is thought to be due to the elevated estrogen levels, which lead to increased cholesterol crystal aggregation and bile viscosity, as well as the effects of progesterone, which induces gallbladder smooth muscle relaxation and bile stasis. These physiological changes may also contribute to the development of gallbladder and biliary tract cancer in women with preeclampsia [13,14]. Several studies have reported an association between gallbladder-related issues and preeclampsia. However, our research is the first to examine the association between preeclampsia and gallbladder and biliary tract cancer. This important and novel finding expands our understanding of the potential long-term health consequences of preeclampsia. However, it must be acknowledged that the wide confidence interval associated with this estimate (HR, 5.49; 95% CI, 1.23–24.50) reflects the inherent rarity of gallbladder and biliary tract cancer in the reproductive-age female population, with only six events observed in the preeclampsia group and five in the control group over the study period. The statistical power is correspondingly limited, and the point estimate should therefore be interpreted with caution. Nonetheless, the finding achieves conventional significance thresholds and is mechanistically plausible, providing a compelling rationale for larger prospective studies specifically designed to validate this association.

Preeclampsia has traditionally been associated with a decreased risk of breast cancer. However, the literature on this topic is inconsistent, and there is a lack of consensus on this issue [15]. Contrary to the findings of previous studies, our results show that preeclampsia is associated with an increased risk of breast cancer. Several factors, including chronic hypertension associated with preeclampsia, usage of antihypertensive medication, and high progesterone levels observed in pregnant women with preeclampsia have been hypothesized to contribute to an increased risk of breast cancer [8,9,10].

In this study, women with preeclampsia were more likely to develop thyroid cancer than the control group. This association has not been previously reported in the literature. Previous studies have suggested an association between subclinical hypothyroidism during pregnancy and an increased risk of preeclampsia. However, the relationship between thyroid cancer and preeclampsia has not been explored [16,17].

Several mechanisms may plausibly link preeclampsia to thyroid carcinogenesis. Pregnancy and preeclampsia are accompanied by alterations in thyroid-stimulating hormone and human chorionic gonadotropin, by shifts in iodine handling [18] and angiogenic-factor balance (including soluble fms-like tyrosine kinase-1 and placental growth factor) [19], and by a heightened systemic inflammatory state, all of which have been implicated in thyroid follicular-cell proliferation [20]. Nonetheless, an important alternative explanation is detection bias. In South Korea, the widespread uptake of thyroid ultrasonography has produced a well-documented epidemic of thyroid cancer overdiagnosis [21], and women with preeclampsia—who undergo more frequent obstetric and postpartum medical contact than women in the control group—may have a correspondingly greater opportunity for incidental detection of small, indolent thyroid carcinomas. A similar surveillance effect could contribute to the elevated breast cancer risk. The observed thyroid and breast associations should therefore be interpreted with this potential ascertainment bias in mind. Finally, the cancers examined here may be inter-related rather than independently linked to preeclampsia: synchronous or metachronous breast and thyroid cancers occur more frequently than expected by chance [22], such that a diagnosis of one may increase the likelihood of detecting the other, which could partly account for the concurrent elevation of breast and thyroid cancer risk in our study population.

Compared to the previously discussed findings, this study observed the protective effects of preeclampsia against carcinogenesis in reducing the risks of ovarian cancer and leukemia. Particularly, preeclampsia was correlated with a reduced risk of ovarian cancer. The relationship between preeclampsia and ovarian cancer is an ongoing debate in the literature. Notably, lower estrogen levels observed in pregnant women with preeclampsia may account for the protective effect against ovarian cancer observed in our study [23]. However, a previous study conducted among Jewish women with preeclampsia found an increased risk of cancer at all sites, including ovarian cancer [24].

According to current guidelines, women with an average breast cancer risk are advised to undergo biennial screening from 50 to 74 years of age. The decision to initiate mammography screening before the age of 50 years should be made on an individual basis. Women who value the potential benefit more than the potential harm may choose to begin screening between ages 40 and 49 years [25]. However, screening asymptomatic adults for gall bladder, biliary tract, and thyroid cancer is not recommended unless they have a high cancer risk due to hereditary factors. Therefore, consideration should be given to initiating early surveillance for breast gall bladder, biliary tract, and thyroid cancers in patients with a history of preeclampsia [26,27].

Our study found a significant association between preeclampsia and a decreased risk of leukemia, which has not been previously reported in the literature. However, it is an intriguing finding that warrants further investigation. The association may be related to leukemia inhibitory factor, a cytokine in the interleukin-6 family, that plays a critical role in pathways related to the pathogenesis of preeclampsia, such as inflammation, endothelial dysfunction, and hypertension. Further research is needed to fully understand the mechanisms underlying the association between malignancies and preeclampsia and its potential implications for diagnosis, treatment, and prevention. Age-stratified sensitivity analyses further revealed that the overall cancer risk conferred by preeclampsia was primarily concentrated in the youngest reproductive age group (20–29 years; adjusted HR, 1.217; 95% CI, 1.055–1.403), whereas no significant association was observed among women aged 30–39 years or 40–49 years. This age-dependent pattern is consistent with the hypothesis that the immunological and endocrine perturbations induced by preeclampsia—including altered angiogenic factor profiles, heightened systemic inflammation, and hormonal dysregulation—may exert a more pronounced carcinogenic influence in younger women, who have a longer postpartum life expectancy during which these biological alterations may accumulate. The thyroid cancer elevation was also disproportionately driven by the 20–29-year age group (incidence rate 131.64 vs. 90.26 per 100,000 person-years; *p* = 0.0003), whereas the protective association with ovarian cancer was most evident among women aged 30–39 years (3.69 vs. 11.00 per 100,000 person-years; *p* = 0.0008), possibly reflecting the greater influence of low estrogen levels on ovarian carcinogenesis at the peak reproductive period.

To our knowledge, this population-based study is the largest to assess the association between preeclampsia and cancer risk. The use of cancer diagnostic codes registered in the HIRA database, which are considered accurate [28], lends credibility to the data and conclusions of our study. However, when interpreting the results, one should be mindful of the study limitations, such as the lack of information on multifetal (twin and higher-order) gestation, parity, menopausal status, medication history, previous hysterectomy or oophorectomy status, and lifestyle variables such as smoking or alcohol consumption, which may influence the incidence of malignancies. Plurality data were also unavailable: although multifetal (twin and higher-order) gestation is a recognized risk factor for preeclampsia and would thus be more common in the preeclampsia group, it has no established independent association with the cancer sites examined here, so residual confounding from this source is unlikely to explain the observed associations. With respect to the direction of potential bias introduced by unmeasured confounders, smoking and alcohol consumption are generally associated with increased cancer risk [29]; given that preeclampsia patients may have lower rates of these behaviors due to the clinical monitoring inherent to pregnancy care, residual confounding from these variables would most likely attenuate rather than amplify the observed elevated cancer hazard ratios, suggesting that the associations identified in this study may represent conservative estimates. Conversely, the potentially higher body mass index and metabolic comorbidity burden in women with preeclampsia could partly explain the elevated breast and thyroid cancer risks; however, the direction of this bias is uncertain given the complex relationships between metabolic factors and specific cancer types [30]. Additionally, the use of appendectomy patients as the reference group, while methodologically justified, introduces an age distribution that differs significantly from the preeclampsia group; the multivariate adjustment for age in the Cox model mitigates but may not fully eliminate this imbalance. Finally, because the analyses were conducted using administrative claims data, misclassification of the preeclampsia diagnosis cannot be entirely excluded, although the requirement for at least two physician visits with the corresponding ICD-10 code is expected to minimize false-positive ascertainment. Participants were followed for a mean of approximately 9.1 years in both groups (corresponding to 383,968 person-years in the preeclampsia group and 953,846 person-years in the control group), which afforded substantial accrual of incident cancers while limiting the ability to characterize long-latency malignancies; because the analyses were conducted within the HIRA closed research environment—from which only pre-specified aggregate outputs (person-years, event counts, and model estimates) could be exported and to which access has since been closed—a patient-level summary of the interval from the index date to cancer diagnosis could not be regenerated for this revision; nonetheless, requiring incident cancer to occur after the index date guarantees the correct temporal sequence, and the near-identical mean follow-up in the two arms (9.06 years each) indicates that the comparison was not distorted by differential observation time. Moreover, because the study population comprised women aged 20–49 years at the index date and was followed for a mean of approximately 9.1 years, the attained age remained relatively young; the design therefore captures cancer risk during the reproductive and early-midlife period rather than lifetime risk, and has limited power to detect long-latency malignancies that predominate at older ages, so longer follow-up will be required to determine whether these associations persist across the full lifespan. Because numerous anatomical sites were examined, no formal correction for multiple comparisons was applied [31]; site-specific associations based on small numbers of events—particularly gallbladder and biliary tract cancer (11 events in total)—may therefore reflect chance findings and should be regarded as exploratory and hypothesis-generating until replicated in independent populations. Age, the dominant confounder in this setting, was included in the multivariable Cox model, and the adjusted estimates accordingly account for the age imbalance between groups; nonetheless, propensity-score matching or weighting would provide a valuable complementary approach in future work [32], and residual confounding from the differing age structure cannot be entirely excluded. A formal test of the proportional-hazards assumption was not reported [33], and detailed reproductive variables such as mode of delivery (caesarean vs. vaginal) were not available in the extracted claims data; these represent additional limitations to be addressed in subsequent studies.

## 5. Conclusions

In this nationwide, population-based study of women of reproductive age, a history of preeclampsia was associated, after adjustment for age and comorbidities, with an increased risk of breast and thyroid cancer and a decreased risk of ovarian cancer and leukemia. The signal for gallbladder and biliary tract cancer was based on very few events and should be regarded as exploratory. Because the analysis is observational, the effect sizes are modest, and detection and surveillance bias and residual confounding cannot be excluded, these associations should be interpreted as hypothesis-generating rather than as evidence of causation, and they require confirmation in independent populations. Accordingly, the present findings are not sufficient to justify a change in cancer-screening policy for women with prior preeclampsia. They do, however, suggest that preeclampsia may be a clinically informative marker of differential long-term cancer risk and provide a rationale for further prospective research into the underlying biological mechanisms and into whether risk-stratified surveillance could ultimately benefit this population.

## Figures and Tables

**Table 1:** Demographics and clinical characteristics of the participants.

Characteristics	Patients with Preeclampsia (*n* = 42,380)	Controls Without Preeclampsia (*n* = 105,327)	*p*-Value
Age, mean ± SD, years	31.67 ± 4.41	33.51 ± 8.38	<0.0001
20–29, *n* (%)	13,185 (31.11)	38,733 (36.77)	_
30–39, *n* (%)	27,221 (64.23)	37,174 (35.29)	
40–49, *n* (%)	1974 (4.66)	29,420 (27.93)	
Comorbidities, *n* (%)			
Diabetes	1398 (3.30)	1231 (1.17)	<0.0001
Hypertension	2854 (6.73)	2440 (2.32)	<0.0001
Hyperlipidaemia	1021 (2.41)	2682 (2.55)	0.1271
COPD	20 (0.05)	104 (0.10)	0.0020
CKD	61 (0.14)	103 (0.10)	0.0160
Liver cirrhosis	9 (0.02)	42 (0.04)	0.0811
Heart failure	40 (0.09)	67 (0.06)	0.0468
Insurance type			<0.0001
NHI, *n* (%)	41,774 (98.57)	102,808 (97.61)	
Medical aid, *n* (%)	606 (1.43)	2519 (2.39)	

SD, standard deviation; COPD, chronic obstructive pulmonary disease; CKD, chronic kidney disease; NHI, National Health Insurance.

**Table 2:** Incidence of cancer.

Type of Cancer	Patients with Preeclampsia (*n* = 42,380)	Controls Without Preeclampsia (*n* = 105,327)	*p*-Value
All cancer	1279 (3.02)	3596 (3.41)	0.0001
Stomach	43 (0.10)	185 (0.18)	0.0010
Colon and rectum	45 (0.11)	207 (0.20)	0.0001
Lung	10 (0.02)	79 (0.08)	0.0003
Breast	312 (0.74)	937 (0.89)	0.0036
Uterus	21 (0.05)	99 (0.09)	0.0067
Ovary	17 (0.04)	117 (0.11)	<0.0001
Leukaemia	7 (0.02)	49 (0.05)	0.0074
Thyroid	619 (1.46)	1386 (1.32)	0.0297
Oral cavity and pharynx	12 (0.03)	23 (0.02)	0.4643
Oesophagus	1 (0.00)	3 (0.00)	>0.999
Anus and anal canal	0 (0.00)	1 (0.00)	>0.999
Liver	8 (0.02)	32 (0.03)	0.2242
Gallbladder and biliary tract	6 (0.01)	5 (0.00)	0.0886
Pancreas	6 (0.01)	31 (0.03)	0.0934
Nasal cavity and larynx	1 (0.00)	2 (0.00)	>0.999
Thorax	2 (0.00)	6 (0.01)	>0.999
Bone	1 (0.00)	7 (0.01)	0.4531
Retroperitoneum and peritoneum	2 (0.00)	10 (0.01)	0.5280
Cervix	79 (0.19)	200 (0.19)	0.8893
Kidney	21 (0.05)	37 (0.04)	0.2057
Bladder	2 (0.00)	8 (0.01)	0.7343
Brain and spinal cord	7 (0.02)	36 (0.03)	0.0719
Hodgkin lymphoma	0 (0.00)	2 (0.00)	>0.999
Non-Hodgkin lymphoma	22 (0.05)	42 (0.04)	0.3147
Multiple myeloma	0 (0.00)	5 (0.00)	0.3306
Other	102 (0.24)	273 (0.26)	0.5225

**Table 3:** Univariate and multivariate Cox proportional hazard analyses of the cancer risk for all sites after adjustment for age, comorbidities, and preeclampsia.

	*Crude HR (95% CI)*	*p-Value*	*Adjusted HR (95% CI)*	*p-Value*
*Age*	1.060 (1.056–1.064)	<0.0001	1.060 (1.056–1.065)	<0.0001
*Diabetes*	1.312 (1.087–1.583)	0.0047	0.979 (0.801–1.196)	0.8350
*Hypertension*	1.516 (1.337–1.718)	<0.0001	1.089 (0.954–1.244)	0.2067
*Hyperlipidaemia*	1.572 (1.356–1.822)	<0.0001	1.138 (0.970–1.337)	0.1135
*COPD*	1.232 (0.513–2.961)	0.6408	0.952 (0.396–2.289)	0.9122
*CKD*	1.560 (0.782–3.116)	0.2072	1.110 (0.550–2.239)	0.7705
*Liver cirrhosis*	1.892 (0.610–5.866)	0.2696	1.295 (0.417–4.019)	0.6545
*Heart failure*	1.218 (0.457–3.246)	0.6934	0.836 (0.313–2.233)	0.7204
*Preeclampsia*	0.884 (0.830–0.943)	0.0002	1.073 (1.003–1.147)	0.0406

HR, hazard ratio; CI, confidence interval; COPD, chronic obstructive pulmonary disease; CKD, chronic kidney disease.

## Data Availability

The datasets used and/or analyzed during the current study are available from the corresponding authors upon reasonable request.

## Supplementary Materials

The following supporting information can be downloaded at https://www.mdpi.com/article/10.3390/cancers18142218/s1, Supplementary Table S1: Age-stratified sensitivity analyses for cancer risk associated with preeclampsia.

## Abbreviations

CI	Confidence interval
HIRA	Health Insurance Review and Assessment Service
HR	Hazard ratio
ICD	International Classification of Diseases
IRR	Incidence rate ratio
NHI	National Health Insurance
