# Supplementary material for: Preeclampsia and Site-Specific Cancer Risk: A Nationwide Population-Based Study
Source: Cancers (Basel). 2026 Jul 9;18(14):2218. doi: 10.3390/cancers18142218 (PMC13406148; doi:10.3390/cancers18142218)
Supplement: Supplementary file 1 [file cancers-18-02218-s001.zip › cancers-4360850-supplementary.pdf]

Table S1. Age-stratified sensitivity analyses for cancer risk associated with preeclampsia.

Table S1. A. Adjusted hazard ratios for overall cancer risk by age subgroup.

Age subgroup	Controls, n	Preeclampsia, n	Crude HR (95% CI)	p	Adjusted HR (95% CI)	p
Overall	105,327	42,380	0.884 (0.830–0.943)	<0.001	1.073 (1.003–1.147)	0.041
20–29 years	38,733	13,185	1.208 (1.050–1.391)	0.008	1.217 (1.055–1.403)	0.007
30–39 years	37,174	27,221	0.965 (0.887–1.051)	0.418	0.950 (0.871–1.036)	0.248
40–49 years	29,420	1,974	0.921 (0.754–1.126)	0.425	0.910 (0.743–1.113)	0.358

HR, hazard ratio; CI, confidence interval. Adjusted models include age, diabetes, hypertension, hyperlipidemia, chronic obstructive pulmonary disease, chronic kidney disease, liver cirrhosis, and heart failure as covariates. p-values highlighted in yellow indicate statistical significance ($p < 0.05$).

Table S1. B. Cancer incidence rates (per 100,000 person-years) by site and age subgroup.

Cancer site	Overall			20–29 years			30–39 years			40–49 years		
	Control	Preeclampsia	p	Control	Preeclampsia	p	Control	Preeclampsia	p	Control	Preeclampsia	p
All cancers	376.97 (364.75–389.49)	333.10 (315.09–351.87)	<0.001	185.30 (171.42–200.01)	224.85 (199.06–253.06)	0.010	384.42 (363.75–405.96)	369.81 (346.08–394.74)	0.370	627.61 (597.63–658.70)	577.53 (470.41–701.75)	0.400
Stomach	19.39 (16.70–22.40)	11.20 (8.10–15.08)	<0.001	5.34 (3.22–8.34)	1.64 (0.20–5.91)	0.028	19.33 (14.91–24.63)	14.33 (9.98–19.93)	0.143	38.56 (31.41–46.85)	34.31 (12.59–74.68)	0.770
Colon and rectum	21.70 (18.84–24.87)	11.72 (8.55–15.68)	<0.001	7.59 (5.00–11.05)	5.72 (2.30–11.79)	0.474	15.76 (11.80–20.61)	12.29 (8.29–17.54)	0.265	48.48 (40.42–57.69)	45.74 (19.75–90.14)	0.870
Lung	8.28 (6.56–10.32)	2.60 (1.25–4.79)	<0.001	1.41 (0.46–3.28)	0.82 (0.02–4.56)	0.568	4.76 (2.72–7.73)	3.28 (1.41–6.46)	0.372	22.14 (16.81–28.62)	5.72 (0.14–31.86)	0.011
Breast	98.23 (92.04–104.72)	81.26 (72.49–90.79)	0.003	29.24 (23.89–35.43)	31.07 (21.99–42.65)	0.753	105.25 (94.57–116.80)	94.60 (82.80–107.62)	0.203	182.86 (166.85–200.00)	245.88 (177.94–331.20)	0.101
Uterus	10.38 (8.43–12.64)	5.47 (3.39–8.36)	0.002	2.25 (0.97–4.43)	4.09 (1.33–9.54)	0.356	9.22 (6.26–13.08)	6.14 (3.44–10.13)	0.180	22.91	5.72 (0.14–31.86)	0.008

Cancer site	Overall			20–29 years			30–39 years			40–49 years		
	Control	Preeclampsia	p	Control	Preeclampsia	p	Control	Preeclampsia	p	Control	Preeclampsia	p
										(17.48–29.48)		
Ovary	12.27 (10.14–14.70)	4.43 (2.58–7.09)	<0.001	8.15 (5.46–11.71)	4.91 (1.80–10.68)	0.196	11.00 (7.75–15.16)	3.69 (1.69–7.00)	<0.001	19.47 (14.50–25.60)	11.44 (1.38–41.31)	0.347
Thyroid	145.29 (137.74–153.15)	161.21 (148.76–174.43)	0.035	90.26 (80.66–100.70)	131.64 (112.09–153.62)	<0.001	163.82 (150.42–178.09)	178.15 (161.80–195.70)	0.194	196.22 (179.62–213.94)	131.52 (83.37–197.34)	0.024
Leukemia	5.14 (3.80–6.79)	1.82 (0.73–3.76)	<0.001	4.22 (2.36–6.96)	0.82 (0.02–4.56)	0.013	3.27 (1.63–5.85)	2.46 (0.90–5.35)	0.563	8.78 (5.57–13.17)	0.00 (0.00–21.09)	<0.001

Values shown as incidence rate (95% confidence interval). p-values highlighted in yellow indicate statistical significance ($p < 0.05$). Incidence rates were estimated using Poisson regression with person-years as offset, adjusted for age and comorbidities.
